# HaCRT1 of *Heterodera avenae* Is Required for the Pathogenicity of the Cereal Cyst Nematode

**DOI:** 10.3389/fpls.2020.583584

**Published:** 2020-11-19

**Authors:** Jing Liu, Huan Peng, Wen Su, Maoyan Liu, Wenkun Huang, Liangying Dai, Deliang Peng

**Affiliations:** ^1^Key Laboratory for Biology and Control of Plant Diseases and Insect Pests, College of Plant Protection, Hunan Agricultural University, Changsha, China; ^2^State Key Laboratory for Biology of Plant Diseases and Insect Pests, Institute of Plant Protection, Chinese Academy of Agricultural Sciences, Beijing, China

**Keywords:** effector, *Heterodera avenae*, calreticulin, endoplasmic reticulum, PTI

## Abstract

Cereal cyst nematodes are sedentary biotrophic endoparasites that secrete effector proteins into plant tissues to transit normal cells into specialized feeding sites and suppress plant defenses. To understand the function of nematode effectors in *Heterodera avenae*, here, we identified a calreticulin protein HaCRT1, which could suppress the cell death induced by Bax when expressed in *Nicotiana benthamiana*. HaCRT1 is synthetized in the subventral gland cells of pre-parasitic second-stage nematodes. Real-time PCR assays indicated that the expression of *HaCRT1* was highest in parasitic second-stage juveniles. The expression of an HaCRT1-RFP fusion in *N. benthamiana* revealed that it was localized in the endoplasmic reticulum of the plant cell. The ability of *H. avenae* infecting plants was significantly reduced when *HaCRT1* was knocked down by RNA interference *in vitro*. *Arabidopsis thaliana* plants expressing *HaCRT1* were more susceptible than wild-type plants to *Pseudomonas syringae*. The induction of defense-related genes, *PAD4*, *WRKY33*, *FRK1*, and *WRKY29*, after treatment with flg22 was suppressed in *HaCRT1*-transgenic plants. Also, the ROS accumulation induced by flg22 was reduced in the *HaCRT1*-transgenic plants compared to wild-type plants. HaCRT1 overexpression increased the cytosolic Ca^2+^ concentration in *A. thaliana*. These data suggested that HaCRT1 may contribute to the pathogenicity of *H. avenae* by suppressing host basal defense.

## Introduction

The cereal cyst nematode (CCN, *Heterodera avenae*), belonging to the genus *Heterodera*, is one of the most economically damaging plant-parasitic nematodes worldwide (Bonfil et al., 2004; Nicol et al., 2007). *H. avenae* infects monocotyledons, i.e., wheat (*Triticum aestivum*), oats (*Avena sativa*), and barley (*Hordeum vulgare*), and causes extensive annual yield losses in Asia, Europe, America, Australia, and North Africa (Meagher, 1977; Nicol et al., 2003; Peng et al., 2009). The success of parasitism relies on its capability to establish the feeding site syncytium and break through the barrier of host immunity. Secreting proteins into host plant tissues is a major event in the plant–nematode interaction (Bellafiore and Briggs, 2010). These secreted proteins, known as effectors, are synthetized in the nematode’s two subventral and one dorsal gland cells, and consequently, are secreted into the host plant tissues through the stylet, a typical hollow needle-like structure (Mitchum et al., 2013). Nematode effectors transform plant cells into specialized feeding sites and counteract plant defenses by modifying cell walls, interfering with signaling, and regulating epigenetics (Hassan et al., 2010; Gheysen and Mitchum, 2011; Hewezi and Baum, 2013).

Plants use a two-layered innate immune system to overcome infection during the evolutionary arms race with pathogens. The first layer is pathogen-associated molecular pattern (PAMP)-triggered immunity (PTI), which is governed by pattern recognition receptors (Jones and Dangl, 2006; Dangl et al., 2013). Perception of highly conserved PAMPs at the cell surface initiates a basal immune response which includes callose deposition, reactive oxygen species (ROS), activation of mitogen-activated protein (MAP) kinases cascade, cytoskeletal remodeling, ion fluxes, changes in phytohormone levels, and rapid induction of defense gene expression (Liu et al., 2014). However, to invade the host successfully, pathogens evolved effectors to break through PTI. Consequently, plants evolved the second line of defense, effector-triggered immunity, which is triggered by archetypical R proteins that directly or indirectly recognize individual effectors (Kandoth and Mitchum, 2013).

Calcium (Ca^2+^) signaling has been documented to contribute to plant immunity pathways (Du et al., 2009; Cheval et al., 2013). Rapid changes in cytosolic Ca^2+^ concentration happen at different stages during the establishment of plant immune responses when plants are invaded by pathogens (Lecourieux et al., 2006). Recognition of flg22 by the FLS2–BAK1 receptor complex activates the phosphorylation of BIK1 which phosphorylates RBOHD, triggering the release of reactive oxygen species. Subsequently, the plasma membrane-located calcium channels CNGC2 and CNGC4 are phosphorylated and activated by BIK1, which causes an increase in the concentration of cytosolic calcium (Chin et al., 2013; Ranf et al., 2014; Tian et al., 2019). Calreticulin (CRT), a Ca^2+^-binding protein, regulates intracellular Ca^2+^ homeostasis and Ca^2+^-dependent signal pathways. Calreticulin is highly conserved in eukaryotic organisms and consists of three distinct structural domains: a globular N-domain, a proline-rich middle (P) domain, and a C-terminal domain with a typical endoplasmic reticulum (ER) retrieval signal (K/H)DEL (Gelebart et al., 2005; Jia et al., 2009). Studies in *Arabidopsis thaliana* indicated that both AtCRT1/2 and AtCRT3 are involved in regulating plant defense against biotrophic pathogens. AtCRT2 regulates endogenous SA biosynthesis via its C-terminal Ca^2+^-binding capacity to suppress defense responses (Qiu et al., 2012b). On the other hand, AtCRT3 is indispensable for the abundance of the pattern-recognition receptor kinase EFR that can specifically recognize bacterial elongation factor (EF)-Tu to elicit PTI (Li et al., 2009). Mi-CRT, a CRT from *Meloidogyne incognita*, is synthesized in the subventral glands of pre-parasitic J2 and secreted into the apoplast of plant tissues (Jaubert et al., 2005). Mi-CRT was shown to be involved in PTI suppression during the interaction between *M. incognita* and the host plant (Jaouannet et al., 2013). In *Radopholus similis*, Rs-CRT was shown to be crucial for the pathogenicity and reproduction of *R. similis* (Li et al., 2015). Also in the pinewood nematode *Bursaphelenchus xylophilus*, silencing of *Bx-crt-1* reduced the nematode’s propagation ability (Li et al., 2011). *AbCRT-1*, isolated from *Aphelenchoides besseyi*, was specifically located in the esophageal gland and was highly expressed in female nematodes. AbCRT1 possibly participates in stress adaptation, reproduction, and behavioral patterns (Feng et al., 2015).

Most characterized and well-known effectors are from *Heterodera schachtii*, *Heterodera glycines*, and *M. incognita*. However, little known is about effectors secreted by *H. avenae*. In this study, we identified a CRT from *H. avenae*, named HaCRT1, that enhances plant susceptibility by suppressing PTI.

## Results

### Sequence Analysis of *HaCRT1* From *Heterodera avenae*

**FIGURE 1:**
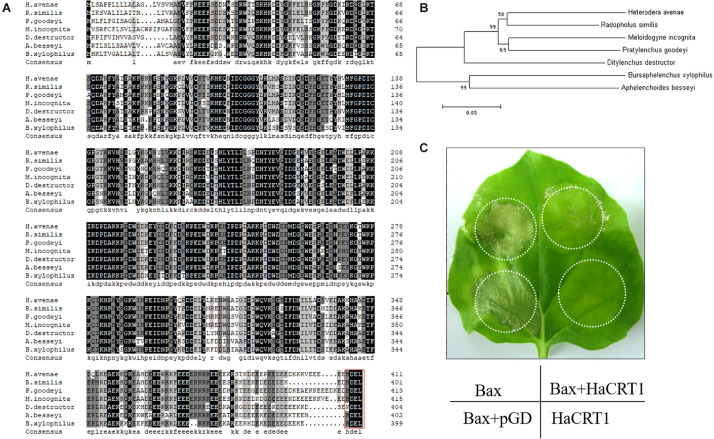
Characterization of HaCRT1. **(A)** Multiple sequence alignment of HaCRT1 with homologs from other plant-parasitic nematodes. Red box shows the ER retrieval signal. **(B)** Phylogenetic tree for HaCRT1 and its homologs from other plant-parasitic nematodes. **(C)** Transient expression of HaCRT1 suppressed cell death induced by Bax.

A series of candidate effectors was identified from transcriptome studies of *H. avenae* to investigate their roles during the interaction with the plant host (Cui et al., 2017). One of those candidate effectors showed high identity with *Mi-CRT* (AAL40720); therefore, it was designated as *HaCRT1*. The sequence of *HaCRT1* (GenBank MT361979) with open-reading frame of 1,236 bp encoded a CRT protein with an N-terminal signal peptide (1–23 aa), a calreticulin region (24–333 aa), and an ER retention site HDEL at the C-terminus. There was no transmembrane domain found in HaCRT1 by prediction of transmembrane helices. Protein BLAST search showed identities of HaCRT1 to CRTs of plant-parasitic nematodes and plants, including *M. incognita* (AAL40720, 80.23% identity), *Pratylenchus goodeyi* (AIW66697, 82.63% identity), *B. xylophilus* (ADD82420, 73.47% identity), *R. similis* (AFK76483, 86.20% identity), *A. besseyi* (AIL52184, 74.26% identity), *Ditylenchus destructor* (ACV33082, 79.89% identity), *T. aestivum* (ABR15365, 54.65% identity), and *A. thaliana* (EFH68751, 56.62% identity) (Figure 1A,B).

### HaCRT1 Suppress BAX-Induced Cell Death

To test the potential role of HaCRT1 when *H. avenae* infects the plant, the coding sequence with the signal peptide was transiently expressed in *Nicotiana benthamiana* under control of the cauliflower mosaic virus CaMV35S promoter. The HaCRT1 did not induce any cell death when it was transiently expressed in *N. benthamiana*. The cell death caused by BAX appeared 2 days after agro-infiltration, but co-expression of BAX and HaCRT1 did not induce cell death even 5 days after agro-infiltration (Figure 1C). This result indicates that HaCRT1 could suppress BAX-induced cell death in *N. benthamiana*.

### HaCRT1 Is Synthesized in the Subventral Esophageal Gland

**FIGURE 2:**
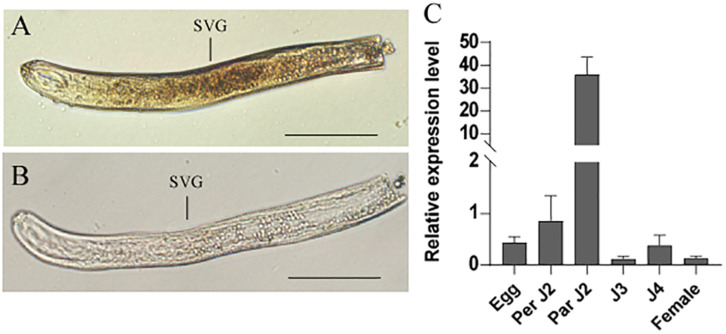
*In situ* hybridization and developmental expression pattern analysis of *HaCRT1*. **(A)** Antisense *HaCRT1* DIG-labeled cDNA probes localized within the subventral glands (SVGs). **(B)** Sense probe as a negative control. Scale bar = 20 μm. **(C)** Developmental expression pattern of *HaCRT1*. The fold change values were calculated using the 2^– Δ Δ *Ct*^ method and presented as the change in mRNA level in various nematode developmental stages relative to that of egg. Each column represents the mean of three independent assays with standard deviation. J2: pre-parasitic second-stage juvenile; parJ2, J3 and J4: parasitic second-, third-, and fourth-stage juvenile, respectively. Three independent experiments were performed with similar results.

*In situ* hybridization was performed to analyze the spatial expression profile of *HaCRT1* in pre-parasitic J2s. The digoxigenin-labeled *HaCRT1* antisense probes were specifically detected in the subventral gland cells (Figure 2A), whereas no signal was detected with the sense probes (Figure 2B), indicating that HaCRT1 was synthesized in the subventral esophageal glands of *H. avenae.* Consequently, to evaluate *HaCRT1* gene expression during *H. avenae* developmental stages, the developmental expression of *HaCRT1* was quantified by qRT-PCR using RNA extracted from six developmental stages. Compared to the female stage, the expression levels of HaCRT1 were higher in pre-parasitic J2 and parasitic J2 stage. In particular, *HaCRT1* transcript accumulated significantly higher in the parasitic J2 stage than in the other stages (Figure 2C). This implies that HaCRT1 plays a role in the early parasitic process of *H. avenae*.

### HaCRT1 Is Targeted to the Endoplasmic Reticulum *in planta*

**FIGURE 3:**
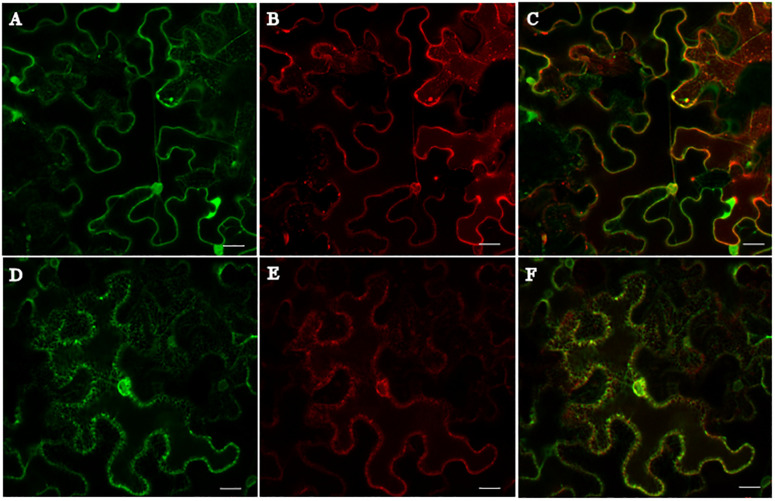
Subcellular localization of HaCRT1. **(A,D)** The GFP signal of endoplasmic reticulum (ER) marker. **(B)** HaCRT1ΔSP fused to RFP. **(E)** HaCRT1 with signal peptide fused to RFP. **(C)** Merged images of HaCRT1ΔSP-GFP and ER-RFP signals. **(F)** Merged images of HaCRT1-GFP and ER-RFP signals. Scale bar = 20 μm.

The subcellular localization of HaCRT1 in plant cells was determined by fusing with fluorescent protein. The HaCRT1 sequence without signal peptide was fused to red fluorescent protein and observed by confocal microscopy imaging. The ER marker (Figure 3A) was co-expressed with HaCRT1ΔSP-RFP (Figure 3B) fusion by injection of transformed *Agrobacterium tumefaciens* cells into *N. benthamiana* leaves. Confocal microscopy showed that HaCRT1ΔSP-RFP signals extensively overlapped with those of the ER marker (Figure 3C), indicating that HaCRT1ΔSP is localized to the endoplasmic reticulum. We also examined the subcellular localization of HaCRT1 with signal peptide. ER marker-GFP (Figure 3D) and HaCRT1-RFP (Figure 3E) fluorescence accumulated in the endoplasmic reticulum, and their co-localization resulted in yellow fluorescence (Figure 3F). The HaCRT1-RFP fusion was observed in the endoplasmic reticulum, which was the same with HaCRT1ΔSP-RFP. Our results indicated that HaCRT1 is primarily localized in the endoplasmic reticulum in plant cells.

### HaCRT1 Increases Susceptibility to DC3000 in *A. thaliana* and RNA Interference of *HaCRT1 in vitro* Impairs *H. avenea* Parasitism

**FIGURE 4:**
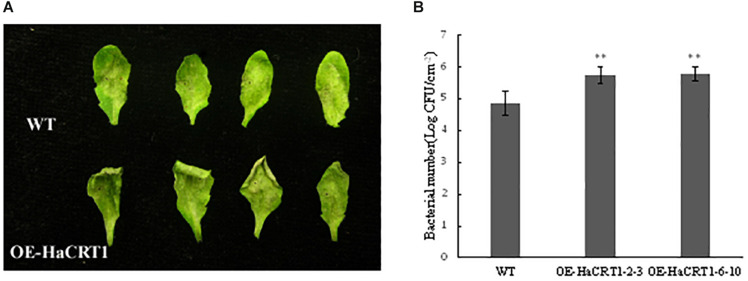
Effect of the overexpression of HaCRT1 on *Arabidopsis thaliana* susceptibility to *Pseudomonas syringae*. **(A)** The necrotic lesions caused by *P. syringae* (*Pto*) pv. *tomato* DC3000 in plants expressing HaCRT1 and WT leaves. **(B)** Bacterial growth of *P. syringae* (*Pto*) pv. *tomato* DC3000 in plants expressing HaCRT1 and WT leaves. Black asterisks indicate a significant difference based on the Student’s *t*-test (**p-value ≤ 0.01). Three independent experiments were performed with similar results.

To gain an insight into the effect of HaCRT1 in plant immunity, transgenic *A. thaliana* constitutively expressing *HaCRT1* full length under the control of the cauliflower mosaic virus 35S promoter were generated. Because *A. thaliana* is not a host of *H. avenae*, we examined whether the expression of HaCRT1 affected the susceptibility of *A. thaliana* to *Pseudomonas syringae* pv. *tomato* DC3000. Compared to wild type, the disease symptoms were more pronounced on leaves of the *HaCRT1* transgenic lines at 3 days after inoculation (Figure 4A) and more bacteria were present (Figure 4B). Those results indicate that *A. thaliana* expressing *HaCRT1* are more susceptible to *Pst* DC3000 than wild-type plants.

**FIGURE 5:**
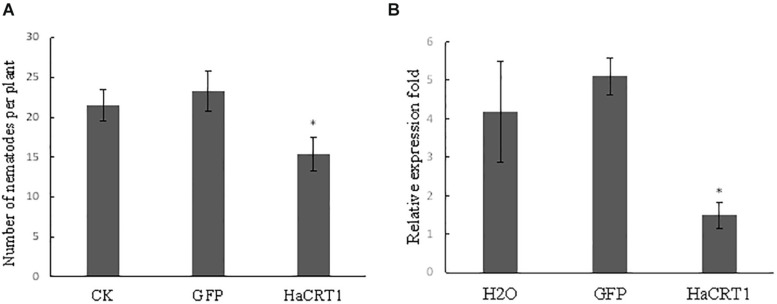
*In vitro* RNAi of *HaCRT1* in *H. avenae*. **(A)** Relative expression level of *HaCRT1* in nematodes treated with *HaCRT1*dsRNAs, *gfp* dsRNA and water. **(B)** The number of nematodes in wheat roots at 10 dpi. Each column represents the mean of three independent assays with standard deviation. Black asterisks indicate a significant difference based on the Student’s *t*-test (*p-value ≤ 0.05).

To investigate the function of HaCRT1 in *H. avenae* parasitism, *HaCRT1* was silenced by soaking nematodes in dsRNA. Real-time PCR analyses showed the mRNA level of *HaCRT1* in nematodes treated with *HaCRT1* dsRNA for 24 h markedly decreased compared with that in nematodes treated with *GFP* dsRNA and water (Figure 5A). *H. avenea* treated with *HaCRT1* dsRNA, *GFP* dsRNA, or water were inoculated on Wenmai 19 to test their infectivity. Compared to water and *GFP* dsRNA treatment, the number of nematodes invaded into roots after *HaCRT1* dsRNA treatment was decreased by 28.5% (Figure 5B). Therefore, HaCRT1 plays an important role in facilitating *H. avenae* parasitism in the early stage.

### HaCRT1 Suppress PTI in *A. thaliana*

**FIGURE 6:**
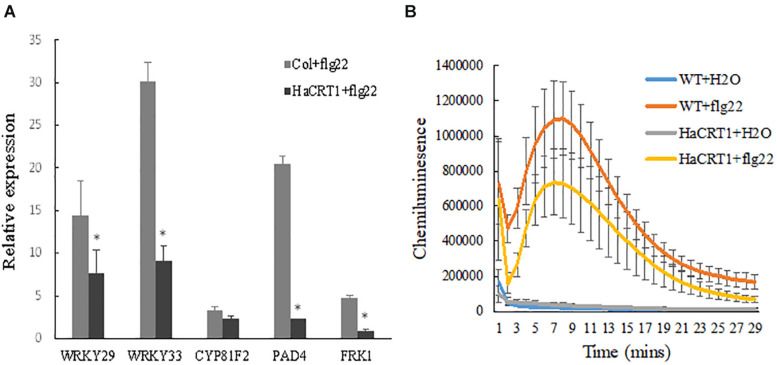
HaCRT1 suppresses PTI in *Arabidopsis thaliana*. **(A)** Expression of defense related genes, WRKY29, WRKY33, PAD4, CYP81F2, and FRK1, was measured by qPCR in *HaCRT1*-transgenic and WT plants treated with flg22. Black asterisks indicate a significant difference based on the Student’s *t*-test (*p-value ≤ 0.05). **(B)** The flg22-mediated ROS production was measured in *HaCRT1*-transgenic and WT plants. The data shown are mean with standard deviation (*p*-value ≤ 0.05). Three independent experiments were performed with similar results.

To investigate whether HaCRT1 regulated plant immunity, real-time PCR was performed to detect the expression of defense-related genes. *PAD4*, *WRKY33*, *FRK1*, and *WRKY29* were strongly induced by flg22 in wild type, but their induction was significantly lower in *HaCRT1*-transgenic plants than in WT plants (Figure 6A). The *HaCRT1*-transgenic leaves were collected to measure ROS generation in response to PAMP treatment. The flg22-triggered ROS burst was remarkedly reduced in *HaCRT1*-transgenic plants compared to WT plants, suggesting that HaCRT1 suppresses PTI in *A. thaliana* (Figure 6B).

### HaCRT1 Interferes With Ca^2+^ Signaling in Plant

**FIGURE 7:**
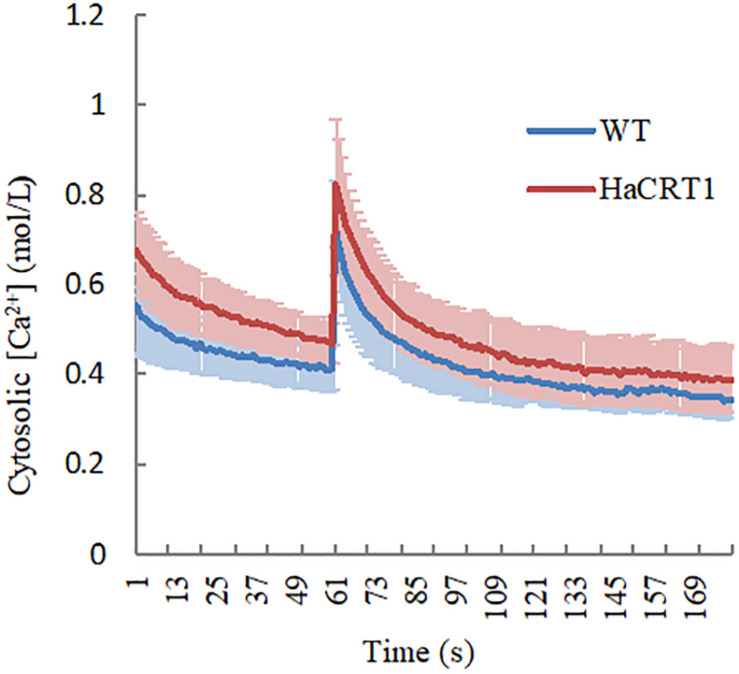
Cytosolic calcium concentration dynamics in *HaCRT1*-transgenic and WT plants. *A. thaliana* [Ca^2+^]_*cyt*_ was assessed by measuring aequorin concentration in response to NaCl treatment. The luminescence was recorded at a 1 s interval. NaCl stimulation was initiated at 60 s. The data shown are mean values ± SEM (*n* = 10) (*p*-value ≤ 0.01). Three independent experiments were performed with similar results.

To test whether HaCRT1 affect [Ca^2+^]_*cyt*_ changes in the plant cell, the calcium reporter aequorin was expressed under the control of the UBQ10 promoter in wild-type and *HaCRT1*-transgenic plants. We treated 10 days old seedlings with NaCl and immediately subjected them to time-based quantitative calcium measurements with a luminometer. Compared with the wild type, *HaCRT1*-transgenic plants displayed higher cytosolic Ca^2+^ concentration before NaCl treatment. When treated with NaCl, there was a transient [Ca^2+^]_*cyt*_ increase of 0.822 ± SEQ 0.143 μM in *HaCRT1*-transgenic plants and 0.725 ± SEQ 0.105 μM in the wild type. *HaCRT1*-transgenic plants showed significant increases in [Ca^2+^]_*cyt*_ than wild type independent on NaCl treatment (Figure 7). These results indicate that HaCRT1 possibly elevates cytosolic Ca^2+^ signaling to mediate plant immunity.

## Discussion

The calreticulin Mi-CRT was first described as an effector in the root-knot nematode *M. incognita*. Mi-CRT is synthesized in the esophageal glands and secreted by the stylet into the apoplasm of host plant tissues and plays an important role during the interaction by suppressing plant basal defenses (Jaubert et al., 2005; Jaouannet et al., 2013). Here, we analyzed the calreticulin HaCRT1 from the cyst nematode *H. avenae* and demonstrated that its role is similar to Mi-CRT in plant-defense suppression. In addition, the HaCRT1 amino acid sequences showed 80.23% sequence identity with the Mi-CRT encoding a highly conserved calreticulin region and an ER retention site HDEL. This study shows that the conserved effector calreticulin plays a similar function in *Meloidogyne* species and *Heterodera*.

Calreticulin, as an abundant Ca^2+^-binding protein, is highly conserved in multi-cellular eukaryotes. So far, CRT have been found from plant-parasitic nematodes including *M. incognita*, *P. goodeyi*, *B. xylophilus*, *R. similis*, *A. besseyi*, and *D. destructor* (Jaubert et al., 2005; Li et al., 2011, 2015; Peng et al., 2013; Feng et al., 2015; Pestana et al., 2015). These CRT proteins contain the calreticulin region and an ER retention site (HDEL) that are highly conserved, and they also have some divergence in foundation and location. Immunolocalizations showed that Mi-CRT was synthesized in the subventral esophageal glands of J2 and the dorsal esophageal gland of sedentary J4 and females (Jaubert et al., 2005). However, AbCRT-1, another calreticulin protein from *A. besseyi*, is specifically located in the esophageal gland and gonads of nematodes (Feng et al., 2015). *Rs-crt* were expressed in the esophageal glands and gonads of females, the gonads of males, the intestines of juveniles, and the eggs of *R. similis* (Li et al., 2015). Our results suggested that *HaCRT1* was expressed in the subventral esophageal glands cells of J2, which is consistent with the location of Mi-CRT. Most nematode effectors are synthesized in the subventral and dorsal esophageal glands during parasitism. The calreticulin was synthesized in the subventral gland cells of *D. destructor* (Peng et al., 2013). *Bx-crt-1*, *AbCRT-1*, and *Rs-crt* were from different species, and they were all highly expressed in female nematodes. However, *HaCRT1* transcript accumulation was significantly highest in the parasitic J2. Those implied that calreticulin from diverse nematodes may have different functions.

As part of the hypersensitive response (HR), cell death is a crucial component in plant immunity (Jamir et al., 2004). Cell death is an ubiquitous event in plant, especially in plant–pathogen interactions. Effector-mediated suppression of cell death resulting from Agrobacterium-mediated transient expression of BAX in *N. benthamiana* leaves has been generally believed as a diagnostic indicator of their function in plant immunity (Chen et al., 2013; Fu et al., 2015). In this study, we found that HaCRT1 could suppress the cell death induced by BAX in *N. benthamiana*, which suggested that HaCRT1 may play a function in plant immunity. We found that the ROS production, which is obviously induced wild-type plants treated with flg22, strongly decreased in *HaCRT1*-transgenic plants treated with flg22. The results from the inoculations, ROS assay, and defense gene expression analysis on HaCRT1-transgenic plants suggest that HaCRT1 may contribute to the virulence of *H. avenae* by suppressing ROS accumulation and plant basal defense. In addition, we detected that the transcript of defense-related genes, *PAD4*, *WRKY33*, *FRK1*, and *WRKY29*, were reduced in *HaCRT1*-transgenic plants. PAD4 is involved in the SA signaling pathway and regulates the accumulation of SA (Rietz et al., 2011). In *FRK1*, as a marker gene of PTI, the mRNA levels could be increased by flg22 treatment (Boudsocq et al., 2010). WRKY29 functions as downstream of the flagellin receptor FLS2, and it is increased in *A. thaliana* after flg22 treatment (Eulgem et al., 2000; Asai et al., 2002). WRKY33 targets the promoter of PAD3 encoding an enzyme required for the synthesis of antimicrobial (Qiu et al., 2008). Thus, we speculated that HaCRT1 may work as a suppressor in plant immunity to facilitate nematode parasitism. It was previously reported that Mi-CRT overexpression *A. thaliana* was more susceptible than wild-type plants to *M. incognita* and *P. parasitica* (Jaouannet et al., 2013). Mi-CRT plays an important role in the suppression of plant immunity defenses during the interaction. Plant CRTs have been shown to play an important role in involving the plant innate immunity, which is usually provoked by plant defense responses against microbial pathogens. NbCRT3a, a calreticulin from *N. benthamiana*, enhanced disease resistance against *Phytophthora infestans* in tobacco (Matsukawa et al., 2013). However, *atcrt1* and *atcrt2* mutant plants showed to be more resistant to the *Pst* DC3000 infection than wild-type plants. AtCRT1 and AtCRT2 negatively regulated plant defense against *Pst* DC3000 in *A. thaliana* (Qiu et al., 2012a). Compared with the wild type, AtCRT2 overexpression plants exhibited more severe disease symptoms after *Pst* DC3000 infection (Qiu et al., 2012b). Therefore, CRT proteins may perform multiple functions in plant defense signaling.

HaCRT1 was found in an ER retention site HDEL at the C-terminus, which usually conferred ER localization. Mi-CRT is localized in the ER and Golgi; however, Mi-CRT without the signal peptide is located in the cytoplasm (Jaouannet et al., 2013). Therefore, the signal peptide was functional and helped Mi-CRT enter the secretory pathway of plants. Consequently, HaCRT1 with and without signal peptide were fused with green fluorescent protein (GFP) reporter to determine where they were localized in plant cell. As we predicted, HaCRT1 without the signal peptide was observed in ER by confocal microscopy imaging. However, HaCRT1 full length was still localized in endoplasmic reticulum. This suggested that the signal peptide of HaCRT1 may not be recognized in *N. benthamiana*. Notably, not all CRTs localized to ER. There are evidences that plant CRT proteins were detected in protein bodies of maize callus storage cells and in plasmodesmata of maize root apex cells (Baluška et al., 1999; Šamaj et al., 2008). Previous studies suggested that the ER stored the amount of calcium ion and regulated Ca^2+^ homeostasis to determine the sensitivity of the cells to apoptotic stress (Joshi et al., 2019). Ca^2+^ is an ubiquitous secondary messenger in cellular signaling involved in developmental processes and abiotic and biotic stresses. The increase of free cytosolic Ca^2+^ is an early indicator and a key event in plant innate immunity against invasive pathogens (Yuan et al., 2017). It has been demonstrated that Ca^2+^ was sensed by CaM, CBL-CIPK pairs, CDPKs, and MAPK activation, then triggered ROS burst and defense-related gene expressions (Seybold et al., 2014). Therefore, we speculated that HaCRT1 may suppress plant defense response by interfering cytosolic Ca^2+^ concentration. To uncover the connections between HaCRT1 and Ca^2+^ signaling, we analyzed the cytosolic Ca^2+^ concentration of *HaCRT1*-transgenic *A. thaliana* and wild type. When *A. thaliana* seedlings were challenged with NaCl, a transient increase in cytosolic Ca^2+^ was observed in both *HaCRT1*-transgenic *A. thaliana* and wild type. Unexpectedly, the cytosolic Ca^2+^ was statistically significantly higher in *HaCRT1*-transgenic plant. *A. thaliana* plants expressing the C-domain of a maize CRT resulted in 9–35% increases of Ca^2+^ accumulation (Wyatt et al., 2002). The ATP-dependent accumulation of Ca^2+^ increased of twofold in tobacco suspension cells overexpressed the maize CRT (Persson et al., 2001). When considering that the elevated levels of CRT enhance the amount of cellular Ca^2+^ stores, it is not surprising that overexpressing HaCRT1 lead to increases of accumulation cytosolic Ca^2+^ in plant cells. Moreover, expression of the AtCRT1 in calreticulin-deficient mouse fibroblasts rescued the phenotype of decreasing levels of Ca^2+^ in the ER (Christensen et al., 2008). Animal CRTs can be substituted by plant CRTs with regard to Ca^2+^ binding, demonstrating that calreticulin basic functions are conserved between across plants and animals. These findings support our hypothesis that the HaCRT1 secreted by nematodes could perform a function in regulating plant defense response by interfering plant cytosolic Ca^2+^ signaling. The *M. incognita* effector MiMIF-2, which led *A. thaliana* more susceptible to nematode infection, impaired Ca^2+^ influx induced by H_2_O_2_. Compared to wild type, *A. thaliana* expressing MiMIF-2 exhibited a decrease in Ca^2+^ influx when stimulated with H_2_O_2_ (Zhao et al., 2019). Here, we found that HaCRT1 suppressed plant immunity and increased the cytosolic Ca^2+^ concentration. Apparently, HaCRT1 involved in plant immunity by regulation of Ca^2+^ signal transduction in a different way than MiMIF-2. However, we do not yet know how HaCRT1 suppress plant defense response by induced cytosolic Ca^2+^ concentration when nematode injected them into plant cell.

In this study, we investigated the calreticulin gene from cereal cyst nematode and demonstrated that the effector protein HaCRT1 was synthesized in the subventral esophageal gland and overcame the immunity defenses to promote cyst nematode parasitism. Furthermore, we speculated that HaCRT1 possibly target the ER where it regulates cytosolic Ca^2+^ store in plant cells. In this study, most data were from *A. thaliana* and *N. benthamiana*. Actually, we tried to obtain HaCRT1-transgenic wheat to perform the experiments, but it failed. We had to use *A. thaliana* to generate the HaCRT1-transgenic plants. Further studies are required to do some experiments with its host plants to understand the function of HaCRT1 in modulating the plant cytosolic Ca^2+^ signaling to influence the plant immunity.

## Materials and Methods

### Nematode Cultivation and Extraction

*H. avenae* were collected from *T. aestivum* cv. Wenmai 19 cultured in a growth chamber as previously described (Liu et al., 2016). The different parasitic stages of *H. avenae* were collected from wheat roots infected with J2s at 5, 10, 20, and 30 days post-inoculation (dpi). Wheat roots were digested at 28°C with shaking at 160 rpm in a 6% cellulose and pectinase solution overnight. The pJ2 were obtained from 5 dpi directly. The J3 and J4 were obtained under the microscope from 20 and 30 dpi separately. Some of the J3 were picked up from 10 dpi. The adult females were got from root surfaces under a dissecting microscope at 40 dpi (De Boer et al., 1999).

### Gene Cloning, Sequence Analyses, and Construction of Plasmid

The coding sequences of *HaCRT1* genes were amplified using the primers HaCRT1F/HaCRT1R and high-fidelity DNA polymerase (TaKaRa, Japan). The homologies of HaCRT1 protein sequence were searched using BLAST against NCBI databases for plant-parasitic nematodes. Signal peptides were predicted using signalP 5.0^1^, and putative transmembrane helices were predicted by TMHMM Server v.2.0^2^. The open-reading frames were predicted by ORF Finder^3^. The sequence homology of the calreticulin proteins was then analyzed using DNAMAN and Clustal X v2.0 software tools.

The full-length sequence of HaCRT1 with the signal peptide was amplified using the primers HaCRT1F (*Pst*I)/HaCRT1R (*Xho*I) transferred to the plant expression vector pYBA1143 and pYBA1137 by restriction enzyme sites (*Pst*I/*Xho*I). HaCRT1 without signal peptide was amplified using the primers HaCRT1NF (*Pst*I)/HaCRT1R (*Xho*I) which were transferred to the plant expression vector pYBA1137 by restriction enzyme sites (*Pst*I/*Xho*I) These constructs were confirmed by sequencing.

### Plant Cultivation and Transformation

*N. benthamiana* plants for agro-infiltration were grown in a growth room at approximately 22°C (14 h light/10 h dark) for 6–8 weeks.

*A. thaliana* (Columbia) seeds were sterilized by soaking for 1 min in ethanol and then for 5 min in 2.63% sodium hypochlorite, and grown on Murashige–Skoog (MS) medium at 25°C (14 h light/10 h dark) a growth chamber.

The plant overexpression construct that contained *HaCRT1* sequences was transformed into *A. tumefaciens* strain GV3101, which were cultured at 28°C at 200 rpm. The bacterial cells were resuspended in 1/2 MS with 5% sucrose, 5 mM MES, 10 μg 6-BA, 200 μL Silwet-77, and pH 5.7, and were adjusted to an OD600 of 0.8. *A. thaliana* bud flowers were soaked in the bacterial suspensions for 10 s for transformation. Transformants of *HaCRT1* were screened MS medium containing 50 mg/L kanamycin. *A. thaliana* plants were moved to soil and grown at 22°C (14 h light/10 h dark).

### *In situ* Hybridization

*In situ* hybridization was performed in *H. avenae* J2s which were fixed with 3% paraformaldehyde at 4°C for 16 h. The digoxigenin (DIG)-labeled sense and antisense cDNA probes were synthesized with the specific primers DsHaCRT1F/DsHaCRT1R by asymmetric PCR (Roche Diagnostics, Mannheim, Germany). The fixed J2s and DIG-labeled sense or antisense probes were incubated at 45°C overnight. Hybridization signals within the nematodes were detected using diluted alkaline phosphatase conjugated antidigoxigenin antibody and were observed with the Olympus IX71 microscope (Long et al., 2013). Three independent experiments were performed.

### RNA Isolation and RT-qPCR Analysis

Total RNA from pre-parasitic J2, parasitic J2, J3, J4, adult females, and eggs was isolated with TRIzol (Invitrogen, Carlsbad, CA, United States). The RNA samples were treated with RNase-free DNase1 to remove DNA contamination, followed by reverse transcription, the SuperScript III First-Strand Synthesis System (TaKaRa, Japan). The primers GAPDH-qS1/GAPDH-qAS1 were internal controls for normalization. The PCR reactions were run using SYBR Premix ExTaq (TaKaRa, Japan) in an ABI Prism 7500 instrument (Applied Biosystems, United States). Relative expression ratios were performed based on the comparative CT method (ΔΔCT) (Livak and Schmittgen, 2002). Primers used in qRT-PCR are listed in Supplementary Table 1. Three independent experiments were performed.

*A. thaliana HaCRT1* transgenic lines 2–3, 6–10, and wild type (Col) were collected after treatment with water control or 100 nM flg22. Total RNA was extracted from frozen seedlings with TRIzol reagent. First-strand cDNA synthesis and RT-qPCR were performed as above described. The expression of the defense marker genes, *FRK1*, *WRKY33*, *WRKY29*, and *PAD4*, was determined by RT-qPCR after 1 h of treatment Ct values were normalized to the Ct value for the Actin gene of *A. thaliana*. Each sample reaction was run in triplicate. Primers used in qRT-PCR are listed in Supplementary Table 1. Three independent experiments were performed.

### Cell Death and Subcellular Localization Analyses

The *A. tumefaciens* strain EHA105 containing recombined constructs were cultured at 28°C at 200 rpm, resuspended with MES buffer (200 μM Acetosyringone, 10 mM MgCl2, and 10 mM MES, pH 5.6), and adjusted to an OD600 of 1.0 and mixed with the RNA-silencing suppressor P19 at 1:1. The mixture was infiltrated into the leaves of *N. benthamiana* grown in a growth room for 6–8 weeks. The empty pYBA1143 vector and pYBA1143:HaCRT1 were infiltrated, respectively. After 24 h, BAX were infiltrated in the same region in the leaves. On the other hand, pYBA1143:HaCRT1and BAX were infiltrated solely as control. Three leaves were infiltrated for each plant, and five plants were performed for each experiment. The cell death could be observed at 48 h after BAX infiltration. Three independent experiments were performed.

The coding sequences of HaCRT1 full length and HaCRT1ΔSP were cloned into the vector pYBA1137 which contained the RFP gene. We used the ER marker created by inserting a synthetic oligonucleotide encoding HDEL at the C-terminus of the GFP genes and adding the signal peptide of AtWAK2 at the N-terminus (Nelson et al., 2007). *A. tumefaciens* carrying pYBA1137:HaCRT1/pYBA1137:HaCRT1ΔSP and ER-marker were allowed to be co-infiltrated into leaves, *N. benthamiana*, as above described. The fluorescence of the fused proteins was observed using laser confocal fluorescence microscopy (Leica TCSSL) 3 days after infiltration. Three independent experiments were performed.

### RNA Interference *in vitro*

The fragment (187–397 bp) of *HaCRT1* was designed as the target. The *HaCRT1* dsRNA and *GFP* dsRNA were synthesized and purified with a MEGAscript RNAi Kit (Applied Bio-systems, Austin, TX, United States) with the primers DsHaCRT1F/DsHaCRT1R and GFPT7F/GFPT7R, respectively. The fresh hatched J2s were soaked in M9 buffer with 50 mM octopamine, 3 mM spermidine, 0.05% gelatin, and 2 mg/mL dsRNA at room temperature and dark for 24 h. Then, the treated J2s were washed with nuclease-free water to remove the external dsRNA. Total RNA was extracted from the treated J2s to detect mRNA expression level of *HaCRT1* by real-time PCR analysis.

The treated J2s were inoculated on 1-week-old *T. aestivum* cv. Wenmai 19 at 100 J2s per plant. Each treated J2s were inoculated on fifteen plans. The roots were harvested at 10 dpi to count the number of nematodes inside roots by acid fuchsin staining. The number of cysts on the infected plant roots was analyzed at 45 dpi inoculation. Three independent experiments were performed.

### *Pseudomonas syringae* Infection Assays

*P. syringae* pv. *tomato* DC3000 strains was cultured on King’ B medium at 28°C. The bacteria were harvested and resuspended with 10 at OD600 of 0.01–0.1 for infiltering in to 4–6 weeks *A. thaliana HaCRT1* transgenic lines 2–3, 6–10, and wild-type leaves. For testing the HR elicited by DC3000, the plants were observed 2 days after infiltering with OD 0.1. For counting the number of bacterial growths, the strains were infiltered with OD 0.01. Three independent experiments were performed.

### ROS Analysis

Leaf disks were cut from 4 to 6 weeks *A. thaliana HaCRT1* transgenic lines 2–3, 6–10, and wild-type plants. Then, the leaf disks were preincubated in sterile distilled water overnight. Three leaf disks were added in the reaction mixture, 100 nM flg22, 1 uL peroxidase-streptavidin (HRP) (Jackson Immunoresearch 016-030-084), 100 uL Immunstar-HRP substrate (Wako Chemicals, Catalog no.120-04891). ROS generation after flg22 treatment in the leaf disks was monitored immediately at 1/10 s for 30 min with GlomaxTM20/20 luminometer (Promega). Three independent experiments were performed.

### Ca^2+^ Concentration Determination

*A. thaliana* UBQ10:Aequorin contained aequorin protein were used for Ca^2+^ concentration measurement as wild type (Ma et al., 2019). *HaCRT1* transgenic lines 2–3, 6–10 crossing with wild type generated F1 plants. The primers AEQ-F/R and HaCRT1F/R were used to identify the homolog recombination plants. The F2 plants contained HaCRT1 and aequorin protein were used for Ca^2+^ concentration measurement. The single 10 days seedling was placed into the 1.5 mL tube and incubated with 20 mmol/L coelenterazine solution (Sigma Catalog no. C3230) at room temperature and dark for at least 4 h. After aequorin reconstitution, the luminescence of seedling was recorded with GlomaxTM20/20 luminometer (Promega). The resting luminescence was measured for 60 s (1 s interval). The 100 mL solution (200 mM NaCl) was added into tube immediately, and the stimulated luminescence was recorded for another 120 s (1 s interval). The 100 mL discharge buffer (1 M CaCl_2_, 20% (*v*/*v*) ethanol) was added to discharge the remaining aequorin and record the signal values for the last 120 s (1 s interval). The quantification of Ca^2+^ concentration (mmol/L) was calculated by the following formula: pCa = 0.332588 (log *k*) + 5.5593, *k* is a rate constant equal to luminescence counts per second divided by total remaining counts (*k* = luminescence counts s^–1^/total luminescence counts remaining) (Rentel and Knight, 2004). At least 10 seedlings for *HaCRT1* transgenic lines and wild type were tested. Three independent experiments were performed.

## Data Availability Statement

The datasets presented in this study can be found in online repositories. The names of the repository/repositories and accession number(s) can be found in the article/Supplementary Material.

## Author Contributions

DP and LD conceived, designed the experiments, and supervised the research. JL analyzed the data and wrote the manuscript. JL, WS, ML, and WH performed the experiments. HP and WH contributed materials. All authors contributed to the article and approved the submitted version.

## Conflict of Interest

The authors declare that the research was conducted in the absence of any commercial or financial relationships that could be construed as a potential conflict of interest.

## Footnotes

1 http://www.cbs.dtu.dk/services/SignalP/

2 http://www.cbs.dtu.dk/services/TMHMM/

3 http://www.ncbi.nlm.nih.gov/projects/gorf/gorf.html
