# Supplementary material for: HaCRT1 of Heterodera avenae Is Required for the Pathogenicity of the Cereal Cyst Nematode
Source: Front Plant Sci. 2020 Nov 19;11:583584. doi: 10.3389/fpls.2020.583584 (PMC7717957; doi:10.3389/fpls.2020.583584)
Supplement: Supplementary file 1 [file Table_1.DOCX]

Supplemental Table1. List of primers in this study.

| HaCRT1F (PstI) | 5’AAAACTGCAG ATGCTTAGTGCTCCCCCACTG 3’ | Cloning HaCRT1 to expression vectors |
| --- | --- | --- |
| HaCRT1NF (PstI) | 5’AAAACTGCAG ATGGAGGTCTTCTTTAAGGAG 3’ |  |
| HaCRT1R (XhoI) | 5’CCGCTCGAG CTAAAGTTCGTCGTGCTCCTCC 3’ |  |
| SH-HaCRT1F | 5’ATCTAAGCACAAAGACGACTACGGG 3’ | HaCRT1 probes for in situ hybridization |
| SH-HaCRT1R | 5’GCGTCGGGGTCCTTGATTTTCTT 3’ |  |
| HaCRT1QF | 5’CGGACAACACTTACGAGGTTCAAAT 3’ | qRT-PCR for HaCRT1 |
| HaCRT1QR | 5’CTTCTTTGCGTCTGGGTCTGGGATA 3’ |  |
| GAPDH-qS1 | 5’AGCGGCACAGAACATCATCC 3’ | Internal control primers for *H. avenae* |
| GAPDH-qAS1 | 5’GGTCCTCCGTGTAGCCCAAA 3’ |  |
| DsHaCRT1F | 5’TAATACGACTCACTATAGGGGATCAAGGCCTGAAAACGAG 3’ | HaCRT1 dsRNA synthesis |
| DsHaCRT1R | 5’TAATACGACTCACTATAGGGACATCACATGGTAAGGCGTC 3’ |  |
| GFPT7F | 5’TAATACGACTCACTATAGGGGCACTACTGGAAAACTACCTG 3’ | GFP dsRNA synthesis |
| GFPT7R | 5’AATACGACTCACTATAGGGGCACGTGTCTTGTAGTTCC 3’ |  |
| Actin F | 5’GGTGTCATGGTTGGTATGGGTC 3’ | Internal control primers for *A. thaliana* |
| Actin R | 5’CCTCTGTGAGTAGAACTGGGTGC 3’ |  |
| WRKY29-F | 5’ATCCAACGGATCAAGAGCTG 3’ | qRT-PCR for WRKY29 |
| WRKY29-R | 5’GCGTCCGACAACAGATTCTC 3’ |  |
| WRKY33-F | 5’GCTGCTATTGCTGGTCACTCC 3’ | qRT-PCR for WRKY33 |
| WRKY33-R | 5’GGTCTCCTCGTTTGGTTCTTCC 3’ |  |
| CYP81F2-F | 5’GTGAAAGCACTAGGCGAAG 3’ | qRT-PCR for CYP81F2 |
| CYP81F2-R | 5’ATCCGTTCCAGCTAGCATCA 3’ |  |
| PAD4-F | 5’GCCGCTTTCACCGCACTTTG 3’ | qRT-PCR for PAD4 |
| PAD4-R | 5’GAGAGATTGGTTTCCGAGCAGAGG 3’ |  |
| FRK1-F | TGCAGCGCAAGGACTAGAG | qRT-PCR for FRK1 |
| FRK1-R | ATCTTCGCTTGGAGCTTCTC |  |
| HaCRT1F | 5’ATGCTTAGTGCTCCCCCACTG 3’ | Identify recombination plants |
| HaCRT1R | 5’CTAAAGTTCGTCGTGCTCCTCC 3’ |  |
| AEQ-F | ATGACAAGCAAACAATACT | Identify recombination plants |
| AEQ-R | TTAGGGGACAGCTCCAC |  |
